# Low-Concentration Indium Doping in Solution-Processed Zinc Oxide Films for Thin-Film Transistors

**DOI:** 10.3390/ma10080880

**Published:** 2017-07-31

**Authors:** Xue Zhang, Hyeonju Lee, Jung-Hyok Kwon, Eui-Jik Kim, Jaehoon Park

**Affiliations:** 1 Department of Electronic Engineering, Hallym University, Chuncheon 24252, Korea; zhangxue00@naver.com (X.Z.); zoozs123@naver.com (H.L.); 2 Department of Convergence Software, Hallym University, Chuncheon 24252, Korea; jhkwon@hallym.ac.kr (J.-H.K.); ejkim32@hallym.ac.kr (E.-J.K.)

**Keywords:** oxide semiconductor, sol-gel precursor, solution process, doping, transistor

## Abstract

We investigated the influence of low-concentration indium (In) doping on the chemical and structural properties of solution-processed zinc oxide (ZnO) films and the electrical characteristics of bottom-gate/top-contact In-doped ZnO thin-film transistors (TFTs). The thermogravimetry and differential scanning calorimetry analysis results showed that thermal annealing at 400 °C for 40 min produces In-doped ZnO films. As the In content of ZnO films was increased from 1% to 9%, the metal-oxygen bonding increased from 5.56% to 71.33%, while the metal-hydroxyl bonding decreased from 72.03% to 9.63%. The X-ray diffraction peaks and field-emission scanning microscope images of the ZnO films with different In concentrations revealed a better crystalline quality and reduced grain size of the solution-processed ZnO thin films. The thickness of the In-doped ZnO films also increased when the In content was increased up to 5%; however, the thickness decreased on further increasing the In content. The field-effect mobility and on/off current ratio of In-doped ZnO TFTs were notably affected by any change in the In concentration. Considering the overall TFT performance, the optimal In doping concentration in the solution-processed ZnO semiconductor was determined to be 5% in this study. These results suggest that low-concentration In incorporation is crucial for modulating the morphological characteristics of solution-processed ZnO thin films and the TFT performance.

## 1. Introduction

Zinc oxide (ZnO) has been extensively studied over the past few decades because it has a large direct band gap energy (3.4 eV), a high exciton binding energy (60 meV), resource availability, and nontoxicity [1,2]. Since its band gap corresponds to UV light (with an approximate wavelength of 365 nm), ZnO is inherently transparent and has an optical transparency of over 85% in the visible light region. Thus, this material can be used in various electronic and optoelectronic devices. Further, ZnO is a well-known binary II–VI semiconductor compound and it is widely used in various ternary and quaternary oxide semiconductors such as zinc tin oxide, indium zinc oxide, and indium gallium zinc oxide. Research is being carried out to improve the electrical characteristics of thin-film transistors (TFTs) fabricated with these oxide semiconductors, and pioneering work has been reported on oxide TFT-driven organic light-emitting diode displays [3,4].

ZnO thin films are usually prepared by various deposition techniques such as atomic layer deposition, pulsed laser deposition, chemical vapor deposition, and sol-gel and sputtering methods [5,6,7,8,9]. Among these, the sol-gel technique has notable advantages over the others, as it offers process simplicity, low-cost manufacturing, and high throughput. This solution-based process is thus believed to be useful for large-area, low-cost manufacture of flexible electronics. However, the performance of solution-processed ZnO TFTs is generally inferior to that of vacuum-processed devices. For example, Brox-Nilsen et al. reported saturation mobility values of over 50 cm^2^/Vs in the ZnO TFTs fabricated by radio-frequency magnetron sputtering at room temperature [10]. Lin et al. reported a saturation mobility of 2–4 cm^2^/Vs in ZnO TFTs processed via spin coating [11]. The most common mobility of solution-processed ZnO TFTs is in the range of 0.1 cm^2^/Vs to several cm^2^/Vs; as a result, low-level integrated circuits of solution-processed TFTs seem adequate for low-end sensors and disposable chips. Such low mobilities of these transistors are intimately associated with the morphological and structural characteristics of solution-processed semiconductor films. Here, the solidification phenomenon in solution-deposited films should be distinguished from the morphological growth in vacuum-deposited films [12]. In vacuum deposition processes, nucleation occurs from the nuclei on the surface of the substrate, allowing sequential bottom-up growth of the film. In contrast, solutes can be accommodated near the bulk and surface of the solution-deposited film while substrate heating causes the solidification to proceed at the bottom. This complexity ultimately leads to the formation of grain boundaries in polycrystalline ZnO thin films deposited through solution processes. Note that grain boundaries in solution-processed ZnO semiconductor films can be a major constraint factor for the TFT performance because they act as trapping centers for mobile charge carriers. Therefore, analyses of the morphological and structural characteristics of solution-processed ZnO films are very important to understand the charge transport behavior of these films and the TFT performance.

The extrinsic doping of ZnO was first investigated in the 1950s [13]. Recent years have seen increased interest in the extrinsic doping phenomenon because high free carrier concentrations and low electrical resistivities can be achieved by the extrinsic doping of ZnO. This is possible because the free carrier concentration and electrical resistivity of ZnO can be controllably engineered by extrinsic doping, which is a prerequisite for its use in electronic devices. Recent studies demonstrated that group IIIB elements such as boron, aluminum (Al), gallium, and indium act as shallow donors in ZnO when they are incorporated on Zn cation sites; the outer p electron of these group IIIB elements is weakly bound in the ZnO lattice, allowing it to be thermally ionized into the conduction band of ZnO [14]. Among these dopants, In-doped ZnO thin films have attracted more focus owing to their superior properties in terms of electrical conductivity, optical transparency, and chemical stability compared to those of undoped ZnO thin films [15,16]. In addition, taking into account the ionic radii, it is clear that In (the ionic radius of In^3+^ is ~80 pm) is best suited to substitute Zn (ionic radius of Zn^2+^ ~ 74 pm) in the ZnO lattice [17,18]. Rokn-Abadi et al. reported better optical and electrical properties of the solution-processed ZnO film by doping it with 2%, 4%, 8% and 16% In concentrations [19]. Singh et al. reported that the minimum electrical resistivity values of the order of 0.045 Ωm could be obtained for solution-processed ZnO films with a 5% In dopant concentration [20]. Biswal et al. reported a reduction in the electrical resistivity of In-doped ZnO films deposited by the ultrasonic chemical spray method, with [In]/[In + Zn] atomic percent ratios of 1.5, 3.0, and 4.0 at. % [7]. These previous studies on In doping in solution-processed ZnO films focused mostly on the electrical, optical, and structural characteristics of the films. Nevertheless, there is still a lack of understanding on the effects of low-concentration In doping on the performance of solution-processed ZnO TFTs. Noteworthy is the significant effect of In doping in ZnO films on the performance of TFTs fabricated using a sputtering system; Cheremisin et al. demonstrated that the low-level In doping of ZnO films is effective to modify the film morphology, thereby contributing to increasing the field-effect mobility and decreasing the threshold voltage of vacuum-processed TFTs [21]. Accordingly, a comprehensive study on the morphological and structural characteristics of In-doped ZnO films deposited by a solution process together with the electrical characteristics of solution-processed In-doped ZnO TFTs is essential for further advancement of this technology.

In this study, we investigated the effects of low-concentration In doping on the chemical and structural characteristics of solution-processed ZnO films. The doping concentration of In in ZnO films was varied from 1–9%. The In-doped ZnO sols and films were analyzed by thermogravimetry coupled with differential scanning calorimetry, X-ray photoemission spectroscopy, X-ray diffraction, and scanning electron microscopy. Bottom-gate/top-contact In-doped ZnO TFTs were fabricated by spin coating, and the electrical characteristics of these TFTs were investigated by analyzing the on/off current ratio and the field-effect mobility. The experimental results demonstrate the significant impact of low-concentration In doping of solution-processed ZnO semiconductor films on the TFT performance.

## 2. Experimental

The In-doped ZnO thin films were prepared by a sol-gel spin coating method. Zinc nitrate hydrate (Zn(NO_3_)_2_·*x*H_2_O) (Sigma-Aldrich, Yongin, Korea) and indium nitrate hydrate (In(NO_3_)_3_·*x*H_2_O) (Sigma-Aldrich) were used as precursors. The atomic percentage of In to Zn was varied from 1% to 9% by varying the stoichiometric quantity of In(NO_3_)_3_·*x*H_2_O with respect to Zn(NO_3_)_2_·*x*H_2_O. The precursor sols were prepared by dissolving the nitrate salts in 2-methoxyethanol (Sigma-Aldrich) at 75 °C for 4 h and filtered through a 0.2 μm polytetrafluorethylene syringe filter.

**Figure 1:**
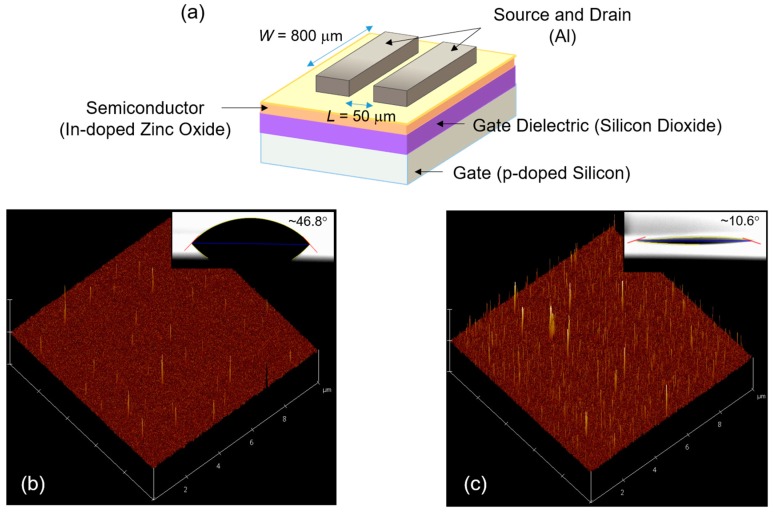
(**a**) Schematic representation of the fabricated In-doped ZnO TFT having the channel length (L) and width (W) of 50 μm and 800 μm, respectively. Atomic force microscope images (10 × 10 μm) of the (**b**) pristine and (**c**) O_2_ plasma-treated SiO_2_ dielectric layers. The insets show the contact angles of both layers.

Figure 1a shows the schematic representation of the fabricated In-doped ZnO TFTs. A heavily boron (p^+^)-doped silicon substrate with a 100-nm-thick silicon dioxide (SiO_2_) dielectric layer (LG Siltron, Seoul, Korea) was used for the fabrication of bottom-gate and top-contact In-doped ZnO TFTs. In order to improve the chemical compatibility of the interface between the dielectric and semiconductor layers we hydrophilized the surface of the SiO_2_ gate dielectric by O_2_ plasma (40 W, 9 SCCM) for 10 s. Figure 1b,c show the surface properties of pristine and O_2_ plasma-treated SiO_2_ dielectric layers, respectively. It is observed that the surface of the SiO_2_ dielectric layer became rougher and more wettable owing to the O_2_ plasma treatment.

The root-mean-square surface roughness values were approximately 10.1 nm for the pristine SiO_2_ layer and approximately 17.7 nm for the O_2_ plasma-treated SiO_2_ layer. From the measured contact angles of the distilled-water drops, the surface energies were calculated to be approximately 51.8 mJ/m^2^ for the pristine SiO_2_ layer and approximately 71.8 mJ/m^2^ for the O_2_ plasma-treated SiO_2_ layer; the surface free energy of distilled water was assumed to be 73.0 mJ/m^2^ [22]. Subsequently, the In-doped ZnO precursor sols were spin-coated on the SiO_2_/Si substrates at a speed of 4000 rpm for 35 s. The coated films were pre-heated on a hot-plate at 120 °C for 5 min and then annealed in a furnace at 400 °C for 40 min. Finally, source and drain electrodes (Al) were thermally deposited on the semiconductor layer through a shadow mask with a channel length (L) and width (W) of 50 μm and 800 μm, respectively.

Thermogravimetric analysis (TGA) (N-1000, Sinco, Seoul, Korea) and differential scanning calorimetry (DSC) (Setaram, DSC 131 Evo, Caluire, France) were performed under a nitrogen atmosphere at a heating rate of 10 °C/min. The chemical characteristics of the In-doped ZnO films were investigated by X-ray photoelectron spectroscopy (XPS) (K-Alpha, Thermo Scientific, Waltham, MA, USA), and the crystallographic properties were characterized by X-ray diffraction (XRD) (DMAX-2500, Rigaku, Tokyo, Japan). The surface morphologies of the films were examined by atomic force microscope (AFM) (XE150, PSIA, Santa Clara, CA, USA) and field-emission scanning electron microscopy (FESEM) (S-4300, Hitachi, Ibaraki, Japan). The electrical characteristics of the TFTs were evaluated using a semiconductor analyzer (4200-SCS, Keithley, Seoul, Korea).

## 3. Results and Discussion

**Figure 2:**
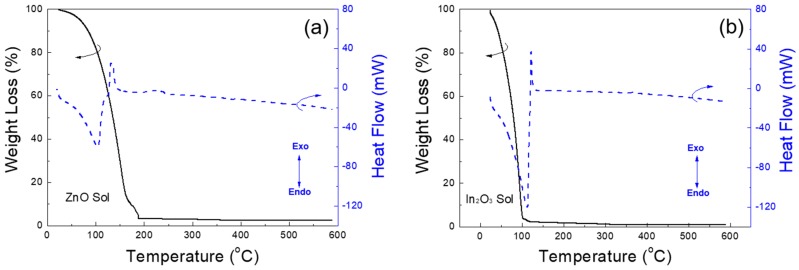

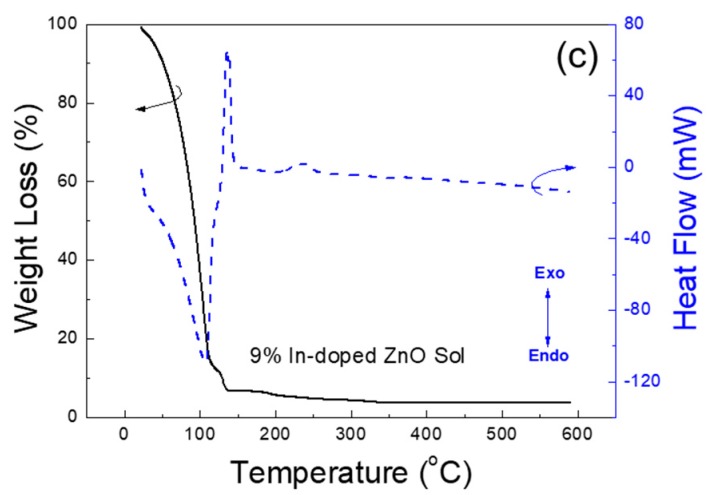
TGA-DSC curves of the (**a**) ZnO; (**b**) In_2_O_3_ and (**c**) 9% In-doped ZnO sols, respectively.

Simultaneous thermal analyses (TGA-DSC) were performed at a scanning rate of 10 °C/min from room temperature to 590 °C under nitrogen, to compare the thermal decomposition of ZnO, indium oxide (In_2_O_3_) and 9% In-doped ZnO sols. Figure 2 shows the TGA-DSC curves of these specimens. From Figure 2, the first endothermic reactions were observed with large weight losses in the range of 25–200 °C. These weight losses are attributed primarily to the low temperature solvent evaporation and precursor decomposition. In these sols, In(NO_3_)_3_·*x*H_2_O and Zn(NO_3_)_2_·*x*H_2_O had decomposed and hydrolyzed to In(OH)_3_ and Zn(OH)_2_, respectively. Because In^3+^ has a larger orbital radius and lower binding energy than Zn^2+^, the temperatures of the first weight loss would decrease with increased indium content [23]. In our results, as shown in Figure 2a–c, the abrupt weight loss continued to the temperatures of approximately 190, 120 and 132 °C for the ZnO, In_2_O_3_, and 9% In-doped ZnO sols, respectively. In the meantime, large exothermic peaks were observed at approximately 150 °C and then the weight loss became gradual. This can be rationalized by the alloying and dehydration of the metal hydroxides to the multi-component oxides [24]. It is well known that the exothermic peaks in the DSC curves with small weight losses correspond to the crystallization of each specimen and the decomposition of the residual organic materials [1]. On the other hand, no meaningful weight losses were detected beyond 400 °C for all the sols. Therefore, we could confirm that an annealing temperature of 400 °C is sufficient to convert the precursors into semiconducting films.

**Figure 3:**
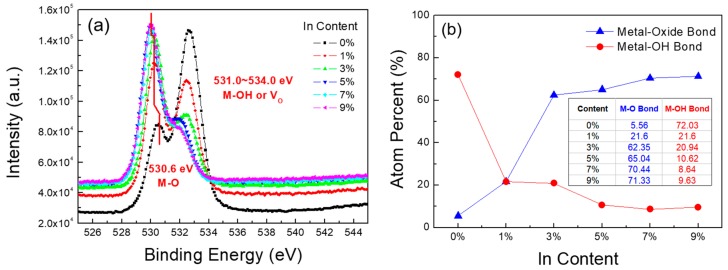
(**a**) O 1s XPS spectra of the In-doped ZnO films with different In concentrations; (**b**) Atom percent of metal-oxide bonds and metal-OH bonds in In-doped ZnO thin films.

X-ray photoelectron spectral analysis was performed to examine the effect of In doping on the chemical-bonding states of the ZnO films. Figure 3a shows the O 1s peaks of ZnO and In-doped ZnO thin films. The O 1s peaks of the In-doped ZnO films shifted slightly toward a lower binding energy, as the In content increased. These peak shifts are attributed to the difference in the bond lengths of In-O (0.210 nm) and Zn-O (0.197 nm), which resulted in the decreases in the binding energy of the O 1s peaks. These peak shifts are consistent with those reported by Martha et al. [25]. The asymmetric peaks observed in the O 1s spectra could be deconvoluted into two peaks. The lower binding energy peaks near 530.6 eV are attributed to the lattice oxygen atoms of the In-O and Zn-O chemical bonds. However, the shoulders in the spectral region between 531.0 and 534.0 eV are attributed to oxygen deficiency and the hydroxide group, which originate from the oxygen vacancy (*V_O_*) and the surface-adsorbed hydroxyl oxygen atoms (M-OH) [26]. Figure 3b shows the results from the deconvoluted XPS spectra of O 1s for the ZnO and In-doped ZnO thin films. Notably, as the In content increased, the quantity of the oxide (M-O) bonds increased from 5.56% to 71.33% and that of the hydroxyl (M-OH) ones decreased from 72.03% to 9.63%, indicating a increase in the fully coordinated metal sites and a decrease in the hydroxyl groups. This is probably due to the additional supply of oxygen atoms from In_2_O_3_, and the difference in the crystallization temperatures of ZnO and In_2_O_3_ thin films, as shown in Figure 2, resulting in the formation of In-doped ZnO thin films under the same annealing temperature.

**Figure 4:**
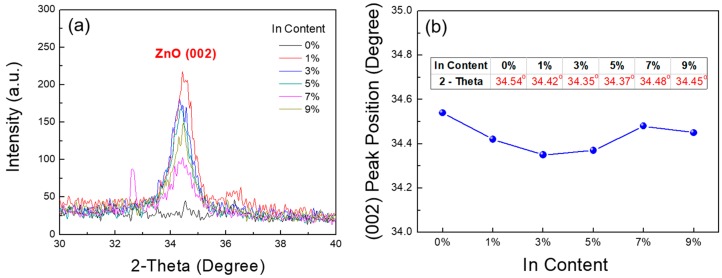
(**a**) XRD patterns and (**b**) the (002) peak positions of the fabricated films with different In contents.

In order to investigate the crystallinity of the In-doped ZnO thin films, the precursor sols with different In contents were spin-coated on SiO_2_/Si substrates and annealed at 400 °C. The XRD in a 2*θ*-scan mode was used to understand the crystalline structure of the fabricated films. As shown in Figure 4a, the (002) diffraction peaks of the ZnO thin films were found with the doping of In, which indicates that In doping improves the crystalline quality of the ZnO thin films. The peaks became broad and the intensity of the (002) diffraction peaks gradually decreased as the In concentration was increased further, which indicates that the crystallinity of the In-doped ZnO films also depends on the elemental content and/or composition of the films. Similar results have been reported by other researchers [25,27]. In Figure 4b, it is observed that all the ZnO (002) diffraction peaks have shifted to smaller angles. This implies that the replacement of Zn^2+^ ions with In^3+^ ions is accompanied by lattice expansion, because the ionic radius of In^3+^ (80 pm) is larger than that of Zn^2+^ (74 pm) [17,18].

**Figure 5:**
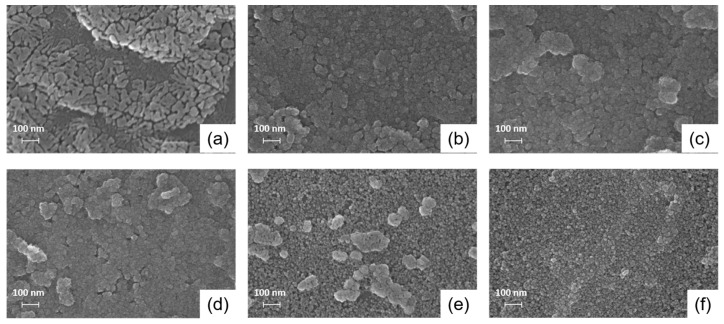
FESEM images of the (**a**) undoped; (**b**) 1%; (**c**) 3%; (**d**) 5%; (**e**) 7% and (**f**) 9% In-doped ZnO films.

Figure 5 shows the top-view FESEM images of the In-doped ZnO thin films with different In contents. As shown in the micrograph (Figure 5a), the surface of the undoped ZnO film shows many cracks. The number of cracks and the roughness of the ZnO films decreased with the increase in the In content. This suggests that In doping has a significant influence on the surface morphology of the solution-processed ZnO film. As the In content increased from 0% to 9%, the grain size of the films decreased obviously. This phenomenon can be attributed to the different ionic radii of the In^3+^ and Zn^2+^ ions. Noteworthy is that the thickness of the In-doped ZnO films increased with the increasing In content up to 5%, and then decreased upon a further increase in the In content. In our results, the thicknesses of the fabricated films were 13.5, 42, 56, 64, 29.7, and 31.1 nm for undoped and 1%, 3%, 5%, 7% and 9% In-doped ZnO thin films, respectively. Likewise, it has also been reported in the literature that the morphology and the crystallinity of the In-doped ZnO films can be changed by the In content. Kim et al. reported that the crystal quality of In-doped ZnO films is enhanced by the incorporation of adequate amounts of In, which is explained by the migration velocity of In being faster than that of Zn and O atoms in the ZnO film due to the In-O bond being weaker than the Zn-O bond [28]. Here, we assume that In^3+^ ions act as Frenkel defects when their content is less than 5% in the primary ZnO structure, indicating that In^3+^ ions place in interstitial sites in the ZnO film. On the other hand, In^3+^ ions prefer to substitute the host Zn atoms and thus act as substitutional impurities when their content exceeds 7%. It is thought that more defect creation energy is required with increasing the In content in the primary ZnO structure, thereby limiting the formation of Frenkel defects in the film. Considering that the ZnO crystal region around an In impurity can be distorted, the lattice possibly becomes more strained around interstitial In^3+^ ions compared to the substitutional In^3+^ ion case; neighboring host atoms will be pushed away if an impurity atom is larger than the host atom [29]. This might cause the increase in the thickness of the In-doped ZnO films with increasing the In content up to 5%. However, the threshold defect creation energy could not be deduced in this study. We expect that further studies using inelastic laser spectroscopy, such as Raman and Brillouin, will be useful to clarify the influence of In content on the energetic state and the distribution of In^3+^ ions in the In-doped ZnO films.

**Figure 6:**
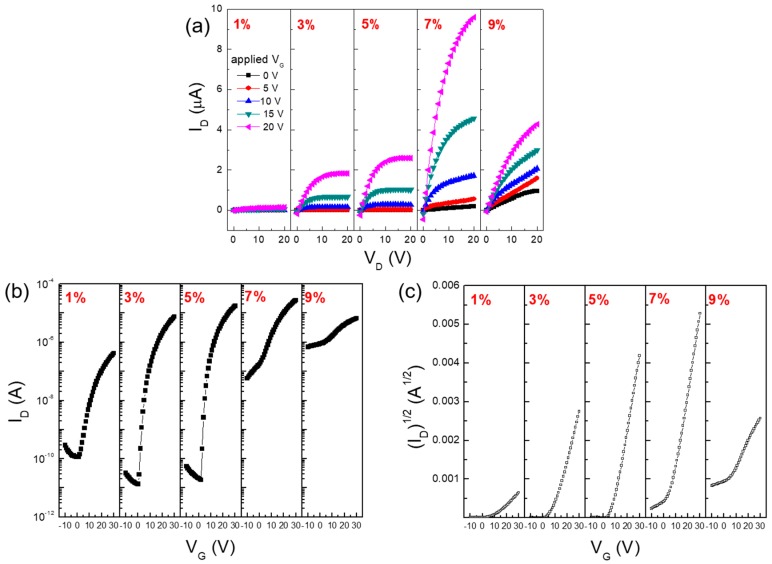
(**a**) *I_D_* vs. *V_D_*; (**b**) *I_D_* vs. *V_G_* and (**c**) (*I_D_*)^1/2^ vs. *V_G_* plots of In-doped ZnO TFTs with different In contents.

To examine the electrical characteristics of the In-doped ZnO thin films, bottom-gate/top-contact ZnO TFTs with SiO_2_/Si substrates were fabricated. In this study, the ZnO TFT did not function as TFTs, even after annealing at 600 °C, due to the very thin nature (approximately 13.5 nm) of the solution-processed ZnO semiconductor film. Note that the electrical resistance of the ZnO semiconductor increased with the decreasing film thickness, and a rather thicker ZnO film is required for TFT operation [30,31]. To emphasize the effect of film thickness on the TFT performance, it will be useful to vary the thickness of solution-processed oxide semiconductor films without altering their chemical and morphological properties. Figure 6a shows the output characteristics of In-doped ZnO TFTs with different In contents. These output characteristics were measured by changing the drain voltage (*V_D_*) from 0 to 20 V in increments of 1 V at different gate voltages (*V_G_*). The drain current (*I_D_*) increased when the In content was increased up to 7% and then decreased when the In content was further increased. Considering the variation in the film thickness according to the In content, the observed change in *I_D_* of In-doped ZnO TFTs is believed to be influenced by the electrical properties of the In-doped ZnO semiconductor films, rather than the effect of the film thickness. Herein, the enhanced conductivities of the In-doped ZnO TFTs could be explained by the larger s orbital of In^3+^, which renders the In-doped ZnO films more conductive, and the increased carrier concentration caused by the addition of the *n*-type dopant, In. Figure 6b,c show the transfer characteristics of In-doped ZnO TFTs with different In contents. These characteristics were measured at a fixed *V_D_* of 15 V, while *V_G_* was swept from –10 to 30 V in increments of 1 V. The electrical properties of the In-doped ZnO TFTs are summarized in Table 1. The on/off current ratio (*I_on_*/*I_off_*) was estimated from *I_D_* vs. *V_G_* plots, as shown in Figure 6b. In our work, the 7% and 9% In-doped ZnO TFTs exhibited remarkably higher off-state currents (exceeding approximately 10^−7^ A) than those (approximately 10^−11^ A) of 3% and 5% In-doped ZnO TFTs. An enhancement in the electrical conductivity, i.e., a reduction in the resistivity, of the 7% and 9% In-doped ZnO semiconductors is the most likely explanation for these higher off-state currents. Note that a back-channel current conduction between source and drain electrodes in bottom-gate/top-contact structured TFTs, which is not controlled by *V_G_*, will dominate the off-state current when the resistivity of the channel layer is too low [32,33]. Previous studies have reported that the ideal resistivity of ZnO films for application in TFTs is about 10^5^–10^6^ Ωcm [34,35]. However, the difference in the on-state currents of these four TFTs seems less significant. As a result, the 5% In-doped ZnO TFT exhibited the highest *I_on_*/*I_off_* value of 9.5 × 10^5^ in this study. For the 1% In-doped ZnO TFT case, it is reasonable to ascribe the lower on-state current to the higher resistance of the 1% In-doped ZnO semiconductor. We consider that a rather high off-state current (approximately 10^−10^ A) of this device is presumably due to deep donor-like states and their energetic distributions in the 1% In-doped ZnO semiconductor [36].

Important TFT parameters such as the threshold voltage (*V_Th_*) and the field-effect mobility (*μ*) were further obtained from the (*I_D_*)^1/2^ vs. *V_G_* plots shown in Figure 6c. Based on the obtained transfer characteristics, *μ* in the saturation region was calculated using the following equation:

(1) $$I_{D}=\frac{W\mu C_{i}}{2L}{\left(V_{G}–V_{Th}\right)}^{2}$$

where *C_i_* is the capacitance per unit area of the gate dielectric layer (34.52 nF/cm^2^). As the In content increased from 1% to 7%, it was clearly observed that *V_Th_* decreased from 11.2 to 4.9 V and *μ* increased from 0.004 to 0.16 cm^2^/Vs. Since the thickness of the 7% In-doped ZnO film is much smaller than those of 1%, 3% and 5% In-doped ZnO films, the enhanced TFT performance should also be interpreted in terms of the increased electron concentration and the enhanced conductivity owing to In doping in a ZnO semiconductor, as exemplified by other groups [23,37]. However, it was meaningless to evaluate the electrical properties of the 9% In-doped ZnO TFT because of the nonsaturating drain current and the floating off-state current, as shown in Figure 6a,b, respectively. The electrical conductivity of the 9% In-doped ZnO film is likely to be detrimental for the gate-field–induced current modulation at the semiconductor/dielectric interface. In this study, the optimal doping level of In in the solution-processed ZnO semiconductor is suggested to be 5% by taking into account the switching and amplification functions of TFTs. Nonetheless, the overall performance of the present 5% In-doped ZnO TFT fabricated by the solution process is still inferior to that of the TFTs in which the In-doped ZnO channel layer was deposited by a vacuum sputtering system [21]. This is possibly due to the rough surface of the O_2_ plasma-treated SiO_2_ dielectric layer and electron trapping at grain boundaries in the solution-processed In-doped ZnO semiconductor films. A nondestructive surface modification, such as the one obtained by the UV/ozone treatment, is expected to afford dielectric layers with a smooth surface [38]. Also, a high-pressure annealing will reduce the generation of defect states in solution-processed oxide semiconductors [39]. Further studies should focus on analyzing the charge transport behaviors in solution-processed oxide TFTs by using different physical models [40,41]. They may help in further enhancing the performance of solution-processed oxide TFTs, as well as in optimizing the material composition.

## 4. Conclusions

We fabricated solution-processed In-doped ZnO films with different concentrations (0%, 1%, 3%, 5%, 7%, and 9%) of the *n*-type dopant, In, using nitrate-based precursors. The XPS analyses of ZnO and In-doped ZnO films showed a shift in the O 1s peak toward a lower binding energy owing to the difference in the bond lengths of In-O and ZnO and an increase in the fully coordinated metal sites and a decrease in the hydroxyl groups. The (002) diffraction peak in the In-doped ZnO films implied an improved crystalline quality of the ZnO film, and its shift toward a lower angle with increasing In content indicated lattice expansion caused by replacing Zn^2+^ ions with In^3+^ ions. These results demonstrate the successful fabrication of In-doped ZnO films with different In concentrations. In addition, the surface morphology and thickness of In-doped ZnO films were significantly affected by the In concentration. A reduction in the cracks and a decrease in the grain size were observed as the In content was increased. An important observation was that the change in the film thickness was not proportional to the In concentration; the thicknesses of the fabricated films were 13.5, 42, 56, 64, 29.7, and 31.1 nm for undoped, 1%, 3%, 5%, 7%, and 9% In-doped ZnO thin films, respectively. Herein, it is assumed that In^3+^ ions act as Frenkel defects when their content is less than 5% in the primary ZnO structure, indicating that In^3+^ ions occupy the interstitial sites in the ZnO film. However, we consider that In^3+^ ions prefer to replace the host Zn atoms and thus act as substitutional impurities when their content exceeds 7%. For the In-doped ZnO TFTs, both the drain current and the field-effect mobility increased, while the threshold voltage decreased as the In content was increased up to 7%. However, the off-state currents of the 7% and 9% In-doped ZnO TFTs showed a large increase of beyond 10^−7^A, causing a significant reduction in the on/off current ratio to below 10^3^. Consequently, the TFT performance can be interpreted in terms of the increased electron concentration and enhanced conductivity owing to In doping in a ZnO semiconductor, rather than the variation in the film thickness. We believe that these results can be useful in analyzing the effect of doping on the structural and electrical properties of solution-processed oxide semiconductors for application in TFTs.

## Figures and Tables

**Table 1:** Electrical characteristics of the In-doped ZnO TFTs with different In contents.

In Concentration (%)	Thickness (nm)	*μ* (cm^2^/Vs)	*V_Th_* (V)	*I_on_/I_off_*
0	13.5	-	-	-
1	42	0.004	11.2	3.5 × 10^3^
3	56	0.07	9.9	5.4 × 10^5^
5	64	0.14	8.4	9.5 × 10^5^
7	29.7	0.16	4.9	5 × 10^2^
9	31.1	-	-	10
